# Early Childhood Growth Parameters in South African Children with Exposure to Maternal HIV Infection and Placental Insufficiency

**DOI:** 10.3390/v14122745

**Published:** 2022-12-09

**Authors:** Mothusi Nyofane, Marinel Hoffman, Helen Mulol, Tanita Botha, Valerie Vannevel, Robert Pattinson, Ute Feucht

**Affiliations:** 1 Department of Consumer and Food Sciences, University of Pretoria, Pretoria 0002, South Africa; 2 Department of Nutrition, National University of Lesotho, Maseru 100, Lesotho; 3 Centre for Maternal, Fetal, Newborn and Child Health Care Strategies, University of Pretoria, Kalafong Provincial Tertiary Hospital, Pretoria 0001, South Africa; 4 Research Unit for Maternal and Infant Health Care Strategies, South African Medical Research Council, Pretoria 0001, South Africa; 5 Department of Paediatrics, University of Pretoria, Pretoria 0002, South Africa; 6 Department of Statistics, University of Pretoria, Pretoria 0002, South Africa; 7 Department of Obstetrics and Gynaecology, University of Pretoria, Pretoria 0002, South Africa

**Keywords:** children who are HIV-exposed-uninfected (CHEU), placental insufficiency, intrauterine growth restriction, child growth

## Abstract

Maternal HIV exposure and intrauterine growth restriction (IUGR) due to placental insufficiency both carry major risks to early child growth. We compared the growth outcomes of children aged 18 months who had abnormal umbilical artery resistance indices (UmA-RI), as a marker of placental insufficiency, with a comparator group of children with normal UmA-RI during pregnancy, as mediated by maternal HIV infection. The cross-sectional study included 271 children, grouped into four subgroups based on HIV exposure and history of normal/abnormal UmA-RI, using available pregnancy and birth information. Standard procedures were followed to collect anthropometric data, and z-scores computed as per World Health Organization growth standards. Lower length-for-age z-scores (LAZ) were observed in children who were HIV-exposed-uninfected (CHEU) (−0.71 ± 1.23; *p* = 0.004) and who had abnormal UmA-RI findings (−0.68 ± 1.53; *p* < 0.001). CHEU with abnormal UmA-RI had lower LAZ (−1.3 ± 1.3; *p* < 0.001) and weight-for-age z-scores (WAZ) (−0.64 ± 0.92; *p* = 0.014) compared to the control group. The prevalence of stunting was 40.0% in CHEU with abnormal UmA-RI and 16.0% in CHEU with normal UmA-RI (*p* < 0.001; *p* = 0.016, respectively). In conclusion, maternal HIV exposure and placental insufficiency are independent risk factors for childhood stunting, with this risk potentiated when these two risk factors overlap.

## 1. Introduction

South Africa (SA) is burdened with a high prevalence (31.6%) of HIV infection in women of childbearing age [1] and pregnant women (30.0%) [2]. Nonetheless, access to antiretroviral therapy (ART) has increased over the years, with a >95% ART coverage during pregnancy and delivery [1], leading to an expanding population of children who are HIV-exposed-uninfected (CHEU). Adverse birth outcomes including intrauterine growth restriction (IUGR) and stillbirths have been documented in women living with HIV (WLHIV) compared to their HIV-negative counterparts, even when on ART [3,4,5,6,7]. IUGR is a clinical term describing a pathological inhibition of fetal growth preventing the fetus from attaining its genetic growth potential [8]. In SA, it is reported that CHEU have similar growth patterns with children who are HIV-unexposed-uninfected (CHUU); however, CHEU have been reported as a high-risk group due to in utero and postnatal ART exposure, as well as exposure to pathogens in immunocompromised family members [9,10].

International literature has indicated that CHEU are more likely than CHUU to experience impaired growth and neurodevelopmental outcomes, even in the context of high maternal ART coverage [11,12,13,14]. In the face of maternal HIV exposure as a risk for poor nutritional status, Black et al. stated that IUGR was associated with postnatal child wasting and stunting [15]. In agreement, Flores-Guillén et al. reported a 21% prevalence of stunting associated with IUGR in the HIV-negative population [7].

Placental insufficiency is one immediate cause of IUGR, which in its extreme can lead to fetal demise/stillbirths [16,17]. IUGR, present in up to 30% of pregnancies, may be the most significant population-based attributable risk factor for preventable stillbirth [17,18]. Research has shown that pre-eclampsia, IUGR, and stillbirths linked to placental insufficiency complicate 10 to 15% of all pregnancies [16]. In developing countries, up to 24% of newborns, approximately 30 million, experience IUGR annually [19]. Low- and middle-income countries (LMICs) carry the highest burden of stillbirths (98%) and perinatal deaths [20]. Children born with IUGR are a high-risk group with short- and long-term morbidity and mortality. IUGR is a crucial risk factor for child undernutrition [21], coupled with an increased risk of overweight and obesity during adolescence, suboptimal intellectual and physical development, and other long-term chronic diseases in adult years [7,22].

The Umbiflow™ device is a low-cost continuous-wave Doppler screening tool for the detection of placental insufficiency in otherwise low-risk pregnancies [23]. Placental insufficiency is detected by an increased umbilical artery resistance index (UmA-RI), also known as an abnormal RI. South African studies have reported a high prevalence of abnormal RI: 11.7% [24]; 13.0% [25]; 5.9% [26].

In view of the above, HIV exposure and placental insufficiency both carry major risks to early and late child growth and development, possibly compounding each other. However, the postnatal growth in children born to otherwise low-risk pregnancies with abnormal RI measurements, indicating IUGR, have not been intensively investigated, particularly in high HIV burden settings. Further, it is well known that adequate child nutrition is central for catch-up growth [27], with a positive correlation between breastfeeding and a child’s growth and development. Exclusive breastfeeding (EBF) stimulates growth among CHEU and CHUU [13], as shown by Jumare et al. Additionally, there is still a lack of evidence on the nutritional management of children born with IUGR to optimize postnatal catch-up growth, including the impacts of feeding practices on the growth of children born with IUGR in the context of HIV exposure.

We therefore investigated and compared, at age 18 months, the growth outcomes of children born with and without IUGR due to placental insufficiency, as measured by an abnormal UmA-RI using Doppler screening during pregnancy, and as modified by maternal HIV status, in the Tshwane District in the Gauteng Province of SA.

## 2. Materials and Methods

### 2.1. Study Settings and Participants

This study followed up participants from the SA arm of the Umbiflow International study, which studied the prevalence of raised UmA-RIs in low-risk pregnant women at 28–34 weeks’ gestation in Ghana, India, Kenya, Rwanda and SA, using a single screening with the Umbiflow™ device between October 2018 and January 2020. Normal and abnormal UmA-RI was defined as <75th centile and ≥75th centile for the gestational age, respectively, as per Pattinson graphs [28]. The mother–child pairs were recruited at 18 months of age into the present study from a prospective longitudinal study and from an additional one-off follow-up from the Umbiflow International study, using the available pregnancy and birth information, at the University of Pretoria’s Research Centre for Maternal, Fetal, Newborn and Child Health Care Strategies, located at Kalafong Provincial Tertiary Hospital, Tshwane District, Gauteng Province, SA. Additional participants were recruited from the Siyakhula study, a longitudinal study on the effects of maternal HIV infection on child outcomes, with an otherwise similar study design and at the same site, to increase the number in the CHEU group with an abnormal UmA-RI. The study population included children who had abnormal UmA-RIs during pregnancy, as a marker for IUGR, compared to a similar group of children with normal UmA-RIs, as mediated by maternal HIV infection.

### 2.2. Study Design

This cross-sectional study explored the growth outcomes of children at the age of 18 months. Exclusion criteria included multiple pregnancies, inability to obtain informed consent, babies born to underage mothers, and babies with chromosomal or structural abnormalities or other severe medical conditions known to impact infant growth and development. The study population was grouped into four subgroups based on HIV exposure and history of normal or abnormal UmA-RI: (1) mothers who are HIV negative with normal UmA-RI (no exposure variable of interest; control group), (2) maternal HIV infection and normal UmA-RI (single exposure), (3) HIV-negative mothers with abnormal UmA-RI (single exposure), and (4) maternal HIV infection with abnormal UmA-RI (double exposure). The outcome variables were child growth parameters. The modifiers were infant feeding practices and other factors known to contribute to child growth; data on potential confounders was collected, including maternal socio-demographic information, medical and obstetric history, nutritional status and lifestyle factors.

### 2.3. Sample Size Determination

**Figure 1:**
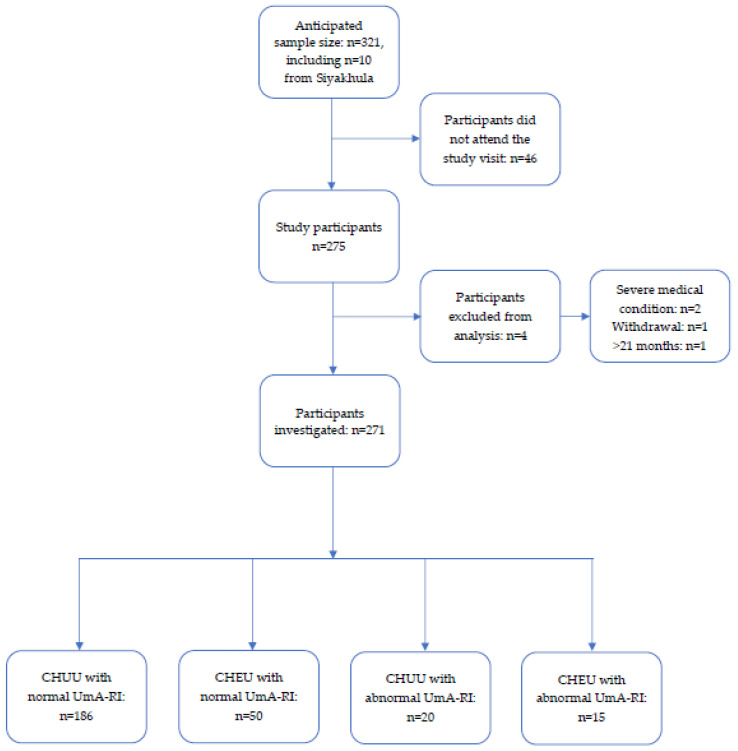
The flow diagram for study participants. Abbreviations: CHEU: children who are HIV-exposed-uninfected; CHUU: children who are HIV-unexposed-uninfected; UmA-RI: umbilical artery resistance index.

The sample size calculation using power analysis indicated that a sample size of 280 was required overall, with a split of 80/20% for CHEU and CHUU. The anticipated sample from the Umbiflow International cohort was 311; however, 46 participants did not attend their study visit, meaning 265 participants were enrolled. Four participants were subsequently excluded either because of age above the set upper limit of 21 months, acute child illness at the study visit or parental choice to not complete the study visit; therefore, this study included 261 participants from the Umbiflow International cohort and an additional 10 participants from the Siyakhula study. The sample size per group for the infant follow-ups was as follows: CHUU with normal UmA-RI: *n* = 186; CHEU with normal UmA-RI: *n* = 50; CHUU with abnormal UmA-RI: *n* = 20; and CHEU with abnormal UmA-RI: *n* = 15 (Figure 1).

### 2.4. Data Collection Methods

Mothers were contacted telephonically and invited to this study, after which written informed consent was obtained by the trained study staff. Data were collected between February and December 2021 using standardized data collection sheets, until no more eligible participants were available due to ageing out. The face-to-face interviews with the mothers were performed in either English or local languages. The child anthropometric measurements collected included weight, length and head circumference (HC), mid-upper arm circumference (MUAC) and triceps skinfold thickness (TSF); similar measurements were collected from the mother except for HC. For intra-observer reliability, the anthropometric measurements, performed as per standard procedures, were taken twice and the mean value was recorded [29,30]. Maternal socio-demographic information was collected using a structured questionnaire, while the descriptive qualitative feeding practices data were collected using a standardized maternal and infant postpartum questionnaire, which is based on adapted World Health Organization (WHO) questionnaires. A structured infant follow-up questionnaire was used to collect maternal and child medical history, along with information obtained from the child’s Road-to-Health booklet, which includes information from clinic visits. For CHEU, HIV antibody testing was performed as per national SA guidelines. One birthweight z-score of >+3 was excluded from the analysis of birth anthropometry measurements (n = 1). Five mothers were pregnant, and a child was brought to the study visit by the caregiver resulting in missing maternal anthropometry data for these mothers.

### 2.5. Data Processing and Statistical Analysis

The data were independently double entered on the online electronic platform, Research Electronic Data Capture (REDCap) v9.3.5, which is a secure web-based application for capturing data in clinical research projects [31]. The outliers were reviewed and corrected in case of error in data capturing, including z-scores outside the range of the reference population (<−3 and >+3). Absolute measurements and z-scores that were clinically implausible were excluded from the analysis. The z-scores for birth anthropometry data were generated using the INTERGROWTH-21^st^ Newborn Size tool (International Fetal and Newborn Growth Consortium for the 21^st^ Century, Oxford, UK) standard version 1.0.6257.25111. There were 12 participants with gestational age (GA) at birth ranging from 43 to 46 weeks, which is clinically unlikely; therefore, the highest GA of 42 weeks 6 days on the INTERGROWTH-21^st^ tool was used for these birth anthropometry z-scores. WHO Anthro software was used to compute the z-scores for the 18-month anthropometric data and children born premature were corrected for gestational age. Both INTERGROWTH-21^st^ and WHO Anthro provide sex- and age-normalized data. This study utilized WHO guidelines to define stunting, wasting, underweight, microcephaly and moderate/severe acute malnutrition as length-for-age z-score (LAZ), weight-for-length z-score (WLZ), weight-for-age z-score (WAZ), HC-for-age z-score (HCAZ), respectively. The z-scores <−3 were classified as severe suboptimal nutritional status. The R statistical program was used for statistical analysis, for which each of the three test groups (with single and double exposure) was compared against the control group to determine if significant differences existed. In all instances, the Shapiro–Wilk test was used to determine if the data were normally distributed. For normally distributed data, the independent t-test was used to compare each test group against the control group, and a one-way ANOVA for comparing all four groups. For the non-normally distributed data, the Mann–Whitney U tests was used to compare each test group against the control group, and the Kruskal–Wallis H test for comparing all four groups. All tests were performed at a 5% level of significance.

### 2.6. Ethical Considerations

Ethical approval for this study was obtained from the Faculty of Natural and Agricultural Sciences and Faculty of Health Sciences Ethics Committees of the University of Pretoria with reference number: NAS259/2021. The mothers were given all the relevant information about the follow-up study prior to recruitment. Mothers provided informed consent on behalf of themselves and their children.

## 3. Results

### 3.1. The Socio-Demographic and Medical Characteristics of the Mothers of the Study Children

A total of 271 mothers were enrolled in this study, with anthropometric measurements not performed in 6 mothers due to repeat pregnancy (n = 5) or because the child was brought by a caregiver (n = 1). The maternal characteristics are presented in Table 1. WLHIV with an abnormal UmA-RI were significantly older (36.6 ± 6.1 years; *p* < 0.001). A high percentage of women had attained any secondary education (80.0%) and 66.7% of WLHIV with abnormal UmA-RI were unemployed. Half of the mothers had access to running water inside the yard and had flushing toilets. More mothers in the control group (29.6%) consumed alcohol at least once a month, compared to other groups. Cigarette smoking was uncommon in all groups. WLHIV with abnormal UmA-RI had lower weight, BMI and MUAC than their counterparts in the other three groups. Further, higher gravidity was reported in WLHIV than their HIV-uninfected counterparts (*p* = 0.018). Two-thirds of women delivered vaginally, but amongst women with history of an abnormal UmA-RI, caesarean section rates were high, with 50.0% of WLHIV and 46.7% of HIV-uninfected women requiring a caesarean section, respectively (Table 1).

### 3.2. The Characteristics of the Study Children

A total of 271 CHUU and CHEU aged 18 months with and without a history of an abnormal UmA-RI in utero were investigated, and the findings are presented in Table 2. The study population had more females than males across the groups, except for CHEU with normal UmA-RI. The GA at birth was lower (36.8 ± 2.5 weeks) in CHUU with abnormal UmA-RI than in the other groups (*p* < 0.001). A history of malnutrition was reported in CHUU with normal UmA-RI (6.0%) and CHEU with normal UmA-RI (12.0%), and a history of diarrhea was common in the study population. Findings on feeding practices showed that CHEU with normal UmA-RI had a lower percentage of early initiation of breastfeeding (within one hour after birth) than the other groups (*p* < 0.001). Lastly, exclusive breastfeeding was lowest in WLHIV with abnormal UmA-RI, while mixed feeding (24.3%) was common in the control group. Percentages of current (supplementary) breastfeeding up to age 18 months were observed to be low in HIV-exposed settings.

### 3.3. Growth Parameters of Study Children

Firstly, investigations involved comparisons of growth outcomes in HIV-exposed vs. unexposed and normal vs. abnormal UmA-RI settings for the entire study population of 271 children (Table 3). When comparing between CHEU and CHUU, lower LAZ was observed in CHEU (−0.73 ± 1.23; ***p*** = 0.003). Similar findings were observed in abnormal UmA-RI when compared to normal UmA-RI group (−0.68 ± 1.53; ***p*** < 0.001).

**Figure 2:**
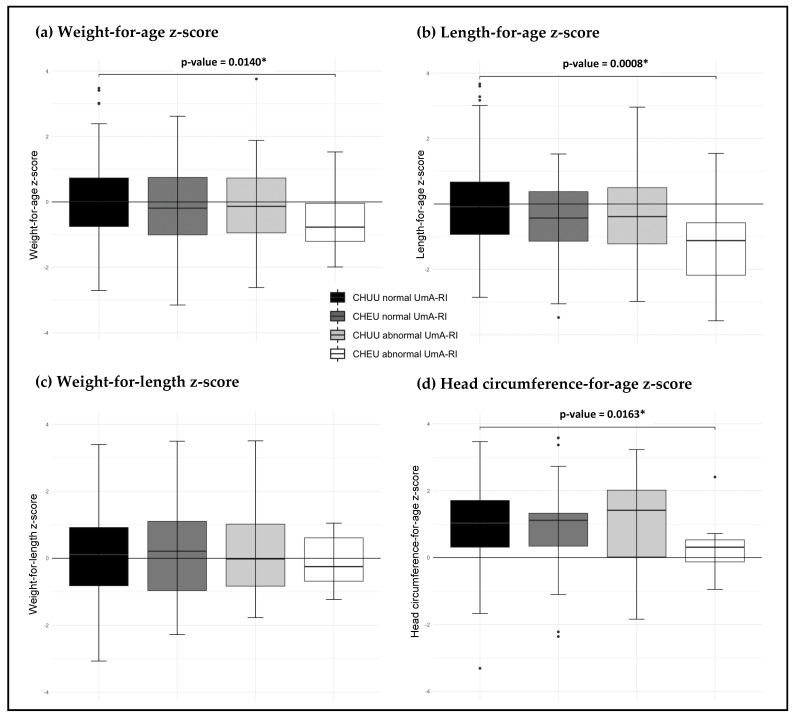
The box plots showing the significant differences for weight-for-age z-score, length-for-age z-score, weight-for-length z-score and HC-for-age z-score between the study groups. The z-scores were computed using World Health Organization (WHO) Anthro software of 2011, using corrected age for premature children. (**a**) WAZ for CHEU abnormal UmA-RI is below the median line and lower than the three groups; (**b**) LAZ for CHEU abnormal UmA-RI is very far below the median and lower than the three groups; (**c**) WLZ for CHEU abnormal UmA-RI is the only group below the median line; (**d**) HCZ for CHEU abnormal UmA-RI is low compared to their counterparts in the different groups. Abbreviations: CHEU: children who are HIV-exposed-uninfected; CHUU: children who are HIV-unexposed-uninfected; UmA-RI: umbilical artery resistance index; WAZ: weight-for-age z-score; LAZ: length-for-age z-score; HCZ: head circumference z-score; HC: head circumference.

Further findings on growth outcomes across the four groups are reported as the means and standard deviations (SD) (Table 4). Investigations involved comparisons of the three test groups against the control group, respectively. CHEU with abnormal UmA-RI had lower WAZ at birth than the control group (***p*** = 0.003). Additionally, lower LAZ was observed in CHEU with normal UmA-RI than the control group (***p*** = 0.023). Findings at 18 months showed that when comparing CHEU with abnormal UmA-RI against the control group, there was a significant difference in weight, length and HC. Further, CHEU with abnormal UmA-RI had significantly lower LAZ (***p*** < 0.001), as well as WAZ and HCZ (***p*** = 0.014; ***p*** = 0.016, respectively) (Figure 2). Furthermore, the findings showed that there were no significant differences for growth outcomes in groups with single exposure, maternal HIV exposure or abnormal UmA-RI, respectively, against the control group. The prevalence of stunting was higher (40.0%) in CHEU with abnormal UmA-RI, than with single exposure, 16.0% in the HIV exposure and abnormal UmA-RI group, respectively (***p*** < 0.001; ***p*** = 0.016). Wasting (8.0%) and underweight (6.0%) were observed in CHEU children with normal UmA-RI. The sensitivity analysis excluding children born preterm showed similar growth outcomes in which CHEU with abnormal UmA-RI had lower LAZ, WAZ and HCZ than the control group: ***p*** = 0.003; ***p*** = 0.029 and ***p*** = 0.029, respectively (data not shown). The rate of stunting remained high among the CHEU with abnormal UmA-RI group (35.7%).

Additional findings indicated that there were no significant differences between the growth indicators and child feeding practices during the first six months of life and up until 18 months of age (Table 5).

## 4. Discussion

Our study shows that infants who had a dual in utero exposure, namely maternal HIV infection and placental insufficiency as measured by an abnormal UmA-RI, had a significantly lower LAZ and higher rates of stunting at 18 months (40.0%), compared to the control group. This finding indicates that maternal HIV infection compounded by unrelated placental insufficiency is an additive risk factor for stunting in SA children. The high percentage of stunting in CHEU contributes to the body of existing knowledge [13,14,32,33,34,35,36], and the finding regarding the high percentage of stunting in CHEU with a history of placental insufficiency and IUGR is novel in the SA context. The prevalence of placental insufficiency and abnormal UmA-RI in SA is high (12%), as reported by Nkosi et al. and Hlongwane et al., and far exceeds numbers previously reported in studies from high-income countries. The etiology is unknown, more so because women included in the SA studies were considered low risk and healthy at the time of screening during their pregnancies.

Lower weight, length and HC reported in the present study among the CHEU-abnormal UmA-RI group at 18 months implies a high-risk group requiring closer follow-up and optimum nutrition care.

Advanced age in WLHIV with abnormal UmA-RI and maternal lifestyle behavior such as use of alcohol were observed in the study population, which have been previously identified as risk factors for placental insufficiency and abnormal UmA-RIs by previous studies [16,37,38]. Adequate mean CD4 T cell counts and low viral loads were observed in the studied WLHIV. A high rate of caesarean section deliveries among the low-risk population has been reported in the Tshwane area, SA [39].

A history of diarrhea was common in the study children, even though most of the mothers self-reported having access to running water and flushing toilets, and diarrhea is known to be a common condition in early childhood. The reported high vertical HIV transmission prophylaxis in the CHEU group points to the success of the prevention of mother to child transmission of HIV (PMTCT) program in SA [40,41,42], with the high percentages of exclusive breastfeeding across the study groups showing improved support and promotion of breastfeeding in SA. Nonetheless, mixed feeding was observed in the present study population, including in CHEU, despite their risk of vertical HIV acquisition. Low adherence to breastfeeding guidelines have been documented in a South African cohort of CHEU and CHUU followed up until 18 months of age [40]. The WHO’s 2016 recommendations on HIV and infant feeding advocate the same breastfeeding practices for all women irrespective of maternal HIV status, within the context of support for adherence to ART for WLHIV [43]. A high stunting rate was observed in the CHEU-abnormal UmA-RI group, with more than half being exclusively breastfed. A systematic review in LMICs stated that limited evidence exist between breastfeeding and growth outcomes [44]. Contrarily, previous studies, including studies from SA, have reported a positive association between exclusive breastfeeding and child growth [13,32,34,45].

Findings on low mean LAZ at 18 months of age in CHEU compared to CHUU counterparts were similar to reports from other African countries, with high stunting rates reported in Ethiopian (27.8%) [46], Nigerian (44.3%) [13] and Kenyan CHEU (20%) [14]. Many other studies have also reported suboptimal growth outcomes in CHEU compared to CHUU [32,33,34,35,36]. Szanyi et al., reported an association between stunting and maternal peri- and postnatal HIV exposure [46]. The present findings differed to those reported by Ramokolo et al. in SA [9] and in Malawi [47], as lower LAZ was observed in CHEU than CHUU. The findings on comparisons of growth outcomes between normal vs. abnormal UmA-RI were similar to those reported in Mexico, which showed a low mean LAZ (−1.22 ± 0.95) and a slightly higher percentage of stunting in participants born with IUGR [7,15,48]. Inadequate growth outcomes in children born with IUGR have been documented in the Philippines and Austria, with a reported association between stunting and IUGR [7,48,49]. According to Stranix-Chibanda et al. (2020), predictive factors for suboptimal growth trajectories, which are most pronounced for LAZ, include IUGR, low birth weight and length [50].

Research on the growth parameters of CHEU with abnormal UmA-RI is limited, with no published reports on the growth outcomes of children exposed to these dual insults. At 18 months of age, CHEU with abnormal UmA-RI had lower mean anthropometric measurements (weight, length and HC) and indicators (WAZ, LAZ and HCZ). The present study population’s normal WLZ and BMI suggest that CHEU with abnormal UmA-RI were symmetrically growth restricted and, as a result, would not be obviously visible within primary healthcare services if not well plotted on growth charts.

A lower mean weight, BMI and MUAC were also observed in the mothers of the CHEU with abnormal UmA-RI, implying that maternal nutrition may negatively influence the child’s linear and ponderal growth. Previous findings indicated that maternal financial situation, BMI, nutrition, education, and age positively correlate with the health of their children [51]. The combination of the dual insult may carry a huge risk of suboptimal child growth, specifically length growth. Advanced age of WLHIV with abnormal UmA-RI may also be attributed to low growth indicators as it may influence childcare and feeding practices. Nevertheless, Fall and colleagues’ findings in a normal population showed that children born to older mothers have less risk of stunting [52].

This study determined and compared the growth outcomes of CHEU with abnormal UmA-RI, a population that was previously unstudied. Study limitations include the small sample size of the subgroups of concern, particularly CHEU with abnormal UmA-RI (5.5%). Further, the high caesarean section rate for a low-risk pregnant population might indicate bias in obtaining a study population born to women with otherwise low-risk pregnancies. The caesarean section rate for the whole Umbiflow-International study (SA arm) group was 28%, which is similar to the rate of 30.1% in this subset. The fact that the children were investigated as a one-off at age 18 months meant that longitudinal data analysis was not possible; therefore, a lack of evaluation of growth over time was a drawback. Additionally, a history of childhood illnesses and breastfeeding practices were based on maternal recall. Future research should investigate CHEU with abnormal UmA-RI over a longer duration in order to better understand their long-term growth trajectories.

## 5. Conclusions

The present study determined and compared the growth outcomes of 18-month-old children born with and without IUGR due to placental insufficiency, modified by maternal HIV status. CHEU with abnormal UmA-RI had lower WAZ, LAZ and HCZ, and are especially at a significantly increased risk of stunting. Maternal HIV exposure and placental insufficiency are independent risk factors for childhood stunting, with this risk potentiated when these two risk factors are compounded.

## Figures and Tables

**Table 1:** The maternal socio-demographic and medical characteristics.

Variables		CHUU with Normal UmA-RI	CHEU with Normal UmA-RI	CHUU with Abnormal UmA-RI	CHEU with Abnormal UmA-RI	*p*-Value ^a^
Sample size (n) (%)		186 (68.6%)	50 (18.5%)	20 (7.4%)	15 (5.5%)	
Mean age (years)		30.1 ± 5.1	31.3 ± 5.5	28.5 ± 4.5	36.6 ± 6.1	<**0.001**
Marital status	Single	79 (42.5%)	21 (42.0%)	6 (30.0%)	4 (26.7%)	n/a
	Married	69 (37.1%)	17 (34.0%)	10 (50.0%)	8 (53.3%)	
	Co-habiting	38 (20.4%)	12 (24.0%)	4 (20.0%)	3 (20.0%)	
Educational level	Any primary schooling	13 (7.0%)	4 (8.0%)	3 (15.0%)	2 (13.3%)	n/a
	Any secondary schooling	127 (68.3%)	40 (80.0%)	13 (65.0%)	12 (80.0%)	
	Post-school education	46 (24.7%)	6 (12.0%)	4 (20.0%)	1 (6.7%)	
Employment status	Unemployed	112 (60.2%)	29 (58.0%)	11 (55.0%)	10 (66.7%)	0.118
	Employed	74 (39.8%)	21 (42.0%)	9 (45.0%)	5 (33.3%)	
Monthly household income ^b^	R 0–R 2000	37 (20.1%)	7 (14.0%)	0 (0%)	4 (26.7%)	n/a
	R 2001–R 4000	43 (23.4%)	15 (30.0%)	8 (40.0%)	0 (0%)	
	R 4001–R 6000	39 (21.2%)	13 (26.0%)	5 (25.0%)	6 (40.0%)	
	R 6001–R 8000	11 (6.0%)	5 (10.0%)	1 (5.0%)	2 (13.3%)	
	R 8000+	45 (24.5%)	9 (18.0%)	6 (30.0%)	1 (6.7%)	
	Don’t know	9 (4.9%)	1 (2.0%)	0 (0%)	2 (13.3%)	
Access to running water	Inside house	68 (36.6%)	10 (20.0%)	7 (35.0%)	4 (26.7%)	n/a
	Inside yard	89 (47.8%)	27 (54.0%)	10 (50.0%)	8 (53.3%)	
	Communal tap	21 (11.3%)	9 (18.0%)	3 (15.0%)	3 (20.0%)	
	Water tank	8 (4.3%)	4 (8.0%)	0 (0%)	0 (0%)	
Access to toilet ^c^	Flushing toilet	131 (70.4%)	30 (61.2%)	16 (80.0%)	9 (64.3%)	n/a
	Pit latrine/bucket	55 (29.6%)	19 (38.8%)	4 (20.0%)	5 (35.7%)	
Drinks alcohol ^d^	Yes	54 (29.2%)	11 (22.0%)	2 (11.1%)	1 (7.1%)	n/a
Smokes cigarettes	Yes	3 (1.6%)	2 (4.0%)	0 (0%)	1 (6.7%)	n/a
Latest CD4 count	Cells/mm^3^ ^e^	N/A	463 ± 310	N/A	416 ± 295	0.147
Latest HIV viral load	Copies/mL (log) ^f,g^	N/A	0.0 [0.0, 4.0]	N/A	0.0 [0.0, 0.0]	0.277
Current ART	TDF/FTC/EFV	N/A	31 (62.0%)	N/A	8 (53.3%)	n/a
	Other ART ^h^	N/A	10 (20.0%)	N/A	6 (40.0%)	
	Not recorded	N/A	9 (18.0%)	N/A	1 (6.7%)	
Obstetric history	Parity ^f^	2 [1, 3]	2 [1, 3]	2 [0, 2]	3 [3, 3]	**0.006**
	Gravidity ^f^	2 [1, 3]	3 [2, 3]	2 [2, 3]	3 [3, 4]	**0.018**
	Previous pregnancy losses ^f^	0 [0, 0]	0 [0, 1]	0 [0, 0]	0 [0, 1]	0.372
	Preeclampsia/eclampsia	2 (13.3%)	0 (0%)	0 (0%)	0 (0%)	n/a
Umbilical artery Doppler	UmA-RI value at 28–34 weeks’ gestation ^g^	0.64 ± 0.05	0.63 ± 0.04	0.74 ± 0.06	0.76 ± 0.04	**<** **0.001**
Mode of delivery	Vaginal delivery	127 (68.3%)	31 (62.0%)	10 (50.0%)	6 (40.0%)	**n**/**a**
	Assisted delivery	3 (1.6%)	0 (0%)	0 (0%)	0 (0%)	
	Caesarean section	56 (30.1%)	19 (38.0%)	10 (50.0%)	9 (60.0%)	
Body measurements and indices ^g,i^	Weight (kg)	77.6 ± 19.6	77.5 ± 25.0	69.7 ± 20.2	63.1 ± 15.4	**0.016**
	Height (cm)	160.2 ± 6.2	161.3 ± 8.9	157.8 ± 5.3	158.3 ± 5.3	0.402
	BMI (kg/m^2^)	30.3 ± 7.7	29.5 ± 8.4	27.9 ± 7.4	25.1 ± 5.2	**0.043**
	MUAC (cm)	32.1 ± 5.0	31.8 ± 5.7	30.7 ± 5.0	28.0 ± 4.0	**0.025**
	TSF (mm)	19.9 ± 5.1	21.4 ± 7.4	20.0 ± 5.3	18.5 ± 3.6	0.243

Values in bold font indicate significant *p*-values. Abbreviations: CHEU: children who are HIV-exposed-uninfected; CHUU: children who are HIV-unexposed-uninfected; UmA-RI: umbilical artery resistance index; CD4: clusters of differentiation 4; ART: antiretroviral therapy; TDF/FTC/EFV: tenofovir/emtricitabine/efavirenz; BMI: body mass index; MUAC: mid-upper-arm circumference; TSF: triceps skin fold; n/a: not applicable. ^a^ Comparisons involved all groups. Only variables with groups above 5 were included in these investigations as smaller groups lead to volatility of results. n/a implies no comparisons were performed due to low counts. ^b^ One South African Rand equates to 0.056 United States Dollars. ^c^ Missing information: CHEU with normal UmA-RI (n = 1); CHEU with abnormal UmA-RI (n = 1). ^d^ Mother drank alcohol at least once in a month, since the baby was born. ^e^ Mean and standard deviation (SD) reported. ^f^ Median and interquartile range [IQR] reported. ^g^ Undetectable viral load is reflected as zero. ^h^ Includes tenofovir (TDF), lamivudine (3TC) plus lopinavir/ritonavir or dolutegravir. ^i^ Total of 265 mothers were measured at the study visit, 5 were pregnant and 1 child was brought by the caregiver.

**Table 2:** The medical background of children from birth to age 18 months.

Variables		CHUU with Normal UmA-RI	CHEU with Normal UmA-RI	CHUU with Abnormal UmA-RI	CHEU with Abnormal UmA-RI	*p*-Value ^a^
Sample size (n) (%)		186 (68.6%)	50 (18.5%)	20 (7.4%)	15 (5.5%)	
Mean age (months)		18.6 ± 0.9	18.5 ± 0.8	18.2 ± 0.2	18.8 ± 0.9	
Sex	Male	91 (48.9%)	28 (56.0%)	8 (40.0%)	4 (26.7%)	n/a
	Female	95 (51.1%)	22 (44.0%)	12 (60.0%)	11 (73.3%)	
Mean GA at birth (weeks)		39.3 ± 1.9	39.4 ± 1.3	36.8 ± 2.5	38.3 ± 1.0	**<** **0.001**
Born premature		15 (8.1%)	2 (4.0%)	7 (35.0%)	1 (6.7%)	n/a
APGAR score (5 min) ^b^		10 [9, 10]	9 [9, 10]	9 [9, 10]	9 [9, 9]	0.069
Neonatal hospitalization		35 (18.9%)	6 (12.0%)	7 (35.0%)	1 (7.1%)	n/a
Neonatal diagnosis ^c^	Respiratory distress	7 (3.8%)	3 (6.0%)	3 (15.0%)	0 (0%)	n/a
	Jaundice	18 (9.7%)	2 (4.0%)	2 (10.0%)	0 (0%)	
	Other	10 (5.4%)	1 (2.0%)	1 (5.0%)	1 (7.1%)	
Prevention of vertical HIV transmission	Single drug (NVP)	N/A	41 (82.0%)	N/A	7 (46.7%)	n/a
	Dual drug (NVP and AZT)	N/A	3 (6.0%)	N/A	6 (40.0%)	
History of childhood illnesses	Malnutrition	11 (6.0%)	6 (12.0%)	0 (0%)	0 (0%)	n/a
	Diarrhea	57 (30.6%)	13 (26.0%)	4 (20.0%)	2 (13.3%)	
Hospital admission (post-neonatal)	Any illness	18 (9.7%)	5 (10.0%)	0 (0%)	1 (6.7%)	n/a
Breastfeeding	Ever breastfeed	178 (95.7%)	47 (94.0%)	20 (100.0%)	15 (100.0%)	
Early initiation of breastfeeding ^d^	Within 1 h after birth	124 (78.5%)	21 (48.8%)	9 (52.9%)	8 (61.5%)	**<** **0.001**
	After 1 h of birth	34 (21.5%)	22 (51.2%)	8 (47.1%)	5 (38.5%)	
Infant feeding from birth until 6 months ^e,f^	Exclusive breastfeeding	122 (65.9%)	32 (64.0%)	14 (70.0%)	8 (53.4%)	0.750
	Formula feeding	11 (5.9%)	3 (6.0%)	0 (0%)	0 (0%)	
	Mixed feeding	45 (24.3%)	8 (16.0%)	4 (20.0%)	2 (13.3%)	
	Formula feeding only at 6 months, but previous exclusive breastfeeding	7 (3.8%)	7 (14.0%)	2 (10.0%)	5 (33.3%)	
Current breastfeeding		50 (27.0%)	3 (6.1%)	4 (22.2%)	2 (14.3%)	n/a

Values in bold font indicate significant *p*-values. Abbreviations: CHEU: children who are HIV-exposed-uninfected; CHUU: children who are HIV-unexposed-uninfected; UmA-RI: umbilical artery resistance index; GA: gestational age; IQR: interquartile range; NVP: nevirapine; AZT: Zidovudine; n/a: not applicable. ^a^ Comparisons involved all groups. Only variables with groups above 5 were included in these investigations as smaller groups lead to volatility of results. n/a implies no comparisons were made due to low counts. ^b^ Median interquartile range [IQR] reported. ^c^ Missing information: CHUU with abnormal UmA-RI (n = 1). ^d^ Unknown information for CHUU with normal UmA-RI (n = 28); CHEU with normal UmA-RI (n = 7); CHUU with abnormal UmA-RI (n = 3); CHEU with abnormal UmA-RI (n = 2). ^e^ Missing information: CHUU with normal UmA-RI (n = 1). ^f^ Comparison was performed for exclusive breastfeeding vs. all other feeding methods.

**Table 3:** The comparisons of mean growth outcomes at age 18 months in HIV-exposed vs. unexposed settings and normal vs. abnormal umbilical artery resistance index (UmA-RI) settings.

Growth Indicators ^a^	CHUU	CHEU	*p*-Value	Normal UmA-RI	Abnormal UmA-RI	*p*-Value
	n = 206 (76.0%)	n = 65 (24.0%)		n = 236 (87.1%)	n = 35 (12.9%)	
WAZ	0.04 ± 1.19	−0.24 ± 1.26	0.122	0.01 ± 1.19	−0.29 ± 1.32	0.122
LAZ	−0.05 ± 1.32	−0.73 ± 1.23	**0.003**	−0.14 ± 1.29	−0.68 ± 1.53	**<** **0.001**
WLZ	0.08 ± 1.21	0.14 ± 1.33	0.710	0.10 ± 1.26	0.04 ± 1.10	0.710
HCZ	0.93 ± 1.18	0.71 ± 1.15	0.198	0.88 ± 1.16	0.85 ± 1.27	0.141

Values in bold font indicate significant *p*-values. Abbreviations: CHEU: children who are HIV-exposed-uninfected; CHUU: children who are HIV-unexposed-uninfected; UmA-RI: umbilical artery resistance index; WAZ: weight-for-age z-score; LAZ: length-for-age z-score; WLZ: weight-for-length z-score; HCZ: head circumference z-score. ^a^ Sex-normalized anthropometric indicators at age 18 months were computed using the World Health Organization (WHO) Anthro software of 2011, corrected for gestational age for preterm infants.

**Table 4:** The mean anthropometric measurements and indicators of study children at birth and age 18 months, as per groups of control, single exposure and dual exposure to maternal HIV plus abnormal UmA-RI.

Variables		CHUU with Normal UmA-RI	CHEU with Normal UmA-RI	CHUU with Abnormal UmA-RI	CHEU with Abnormal UmA-RI	*p*-Value ^a^
Sample size (n) (%)		186 (68.6%)	50 (18.5%)	20 (7.4%)	15 (5.5%)	
**At birth**						
Anthropometry (mean ± SD)	Weight (g)	3187 ± 483	3108 ± 433	2649 ± 566	2704 ± 408	**<** **0.001 ^b^**
	Length (cm)	50.7 ± 3.0	49.9 ± 2.3	48.8 ± 2.9	49.1 ± 2.6	**0.005 ^b^**
	HC (cm)	34.5 ± 1.7	34.5 ± 1.5	33.0 ± 1.9	33.9 ± 1.5	**0.003 ^b^**
Indicators (mean ± SD) ^d^	WAZ	−0.30 ± 1.16	−0.54 ± 0.96	−0.48 ± 1.07	−1.04 ± 0.76	**0.003**
	LAZ	0.68 ± 1.66	0.15 ± 1.32	0.64 ± 1.24	0.36 ± 1.28	**0.023 ^c^**
	HCZ	0.40 ± 1.34	0.35 ± 1.24	0.20 ± 0.95	0.48 ± 1.07	0.778
**At age 18 months**						
Anthropometry (mean ± SD)	Weight (kg)	10.9 ± 1.5	10.7 ± 1.8	10.8 ± 1.9	9.9 ± 1.0	**0.079**
	Length (cm)	81.9 ± 3.8	80.6 ± 3.3	81.1 ± 4.1	78.2 ± 3.5	**<** **0.001**
	HC (cm)	48.1 ± 1.6	48.1 ± 1.9	48.5 ± 1.9	47.1 ± 1.2	**0.011**
	MUAC (cm)	16.0 ± 1.4	16.2 ± 1.7	16.4 ± 1.7	16.0 ± 1.4	0.825
	TSF (mm)	8.7 ± 2.0	8.4 ± 2.2	8.7 ± 2.7	8.6 ± 1.3	0.869
Indicators (mean ± SD) ^e^	WAZ	0.05 ± 1.15	−0.11 ± 1.32	−0.02 ± 1.52	−0.64 ± 0.92	**0.014**
	LAZ	−0.03 ± 1.30	−0.56 ± 1.16	−0.21 ± 1.53	−1.30 ± 1.32	**<** **0.001**
	WLZ	0.07 ± 1.20	0.19 ± 1.46	0.09 ± 1.32	−0.02 ± 0.76	0.662
	HCZ	0.89 ± 1.15	0.83 ± 1.23	1.26 ± 1.47	0.33 ± 0.73	**0.016**
Nutritional classifications (n; %)	Underweight	4 (2.2%)	3 (6.0%)	1 (5.0%)	0 (0.0%)	n/a
	Stunting ^f^	9 (4.8%)	8 (16.0%)	2 (10.0%)	6 (40.0%)	**<** **0.001**
	Wasting	6 (3.2%)	4 (8.0%)	0 (0.0%)	0 (0.0%)	n/a

Values in bold font indicate significant *p*-values. Abbreviations: CHEU: children who are HIV-exposed-uninfected; CHUU: children who are HIV-unexposed-uninfected; UmA-RI: umbilical artery resistance index; HC: head circumference; MUAC: mid-upper-arm circumference; TSF: triceps skinfold; WAZ: weight-for-age z-score; LAZ: length-for-age z-score; WLZ: weight-for-length z-score; HCZ: head circumference z-score; n/a: not applicable. ^a^ Comparisons between CHEU with abnormal UmA-RI group vs. control group. Only variables with groups above 5 were included in these investigations as smaller groups lead to volatility of results. n/a implies no comparisons were made due to low counts. ^b^ Comparisons were made between the four study groups. ^c^ Comparison between CHUU with normal UmA-RI vs. CHEU with normal UmA-RI groups. ^d^ The birth sex-normalized anthropometric indicators were computed using INTERGROWTH-21^st^ software, using gestation-adjusted age for preterm infants. ^e^ The sex-normalized anthropometric indicators at age 18 months were computed using World Health Organization (WHO) Anthro software of 2011, using gestation-adjusted age for preterm infants. ^f^ Additionally, comparison between CHUU with normal UmA-RI vs. CHEU with normal UmA-RI: *p* = 0.016.

**Table 5:** Comparison of mean child growth indicators between breastfeeding practices.

	Feeding Practices during the First Six Months of Life					Continued Breastfeeding at Age 18 Months		
	Exclusive Breastfeeding	Formula Feeding	Mixed Feeding	Formula Feeding Only, but Previously Exclusive Breastfeeding	*p*-Value ^a^	Supplementary Breastfeeding	No Supplementary Breastfeeding	*p*-Value ^a^
Sample size ^b^	176 (65.2%)	14 (5.2%)	59 (21.9%)	21 (7.8%)		59 (21.8%)	212 (78.2%)	
WAZ ^c^	−0.13 ± 1.18	0.03 ± 1.23	0.31 ± 1.25	−0.14 ± 1.24	0.105	0.03 ± 1.25	−0.04 ± 1.20	0.672
LAZ ^c^	−0.34 ± 1.25	0.28 ± 1.33	0.10 ± 1.46	−0.32 ± 1.55	0.067	−0.01 ± 1.46	−0.27 ± 1.29	0.225
WLZ ^c^	0.04 ± 1.19	−0.18 ± 1.78	0.34 ± 1.29	0.01 ± 1.13	0.335	0.05 ± 1.21	0.10 ± 1.25	0.768
HCZ ^c^	0.86 ± 1.17	0.83 ± 1.12	1.07 ± 1.07	0.60 ± 1.39	0.506	1.04 ± 1.10	0.83 ± 1.19	0.220

Abbreviations: WAZ: weight-for-age z-score; LAZ: length-for-age z-score; WLZ: weight-for-length z-score; HCZ: head circumference z-score Sex-normalized anthropometric indicators at age 18 months were computed using World Health Organization (WHO) Anthro software of 2011, using gestation-adjusted age for preterm infants. ^a^ Comparison between the study groups. ^b^ Sample size in numbers and percentages (%). ^c^ Growth indicators are reported as the means and standard deviations (SD).

## Data Availability

Data are available on request from the corresponding author, due to the University of Pretoria policy on data publication.
